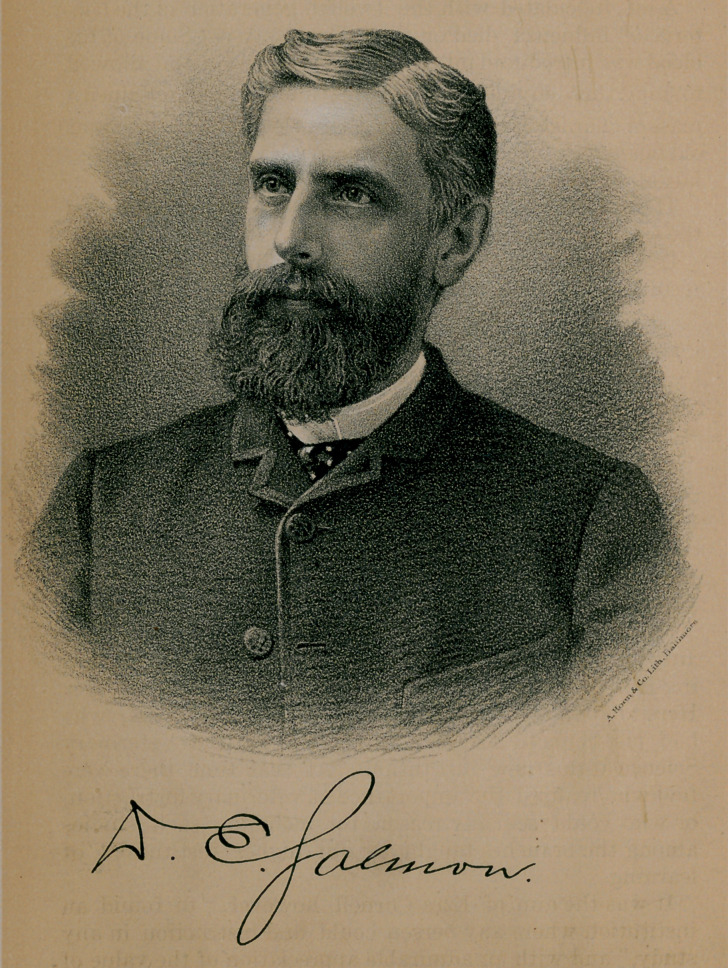# D. E. Salmon, with Portrait

**Published:** 1887-07

**Authors:** 


### Art. XXVII.—DANIEL E. SALMON, D. V. M.

### CHIEF OF THE BUREAU OF ANIMAL INDUSTRY.

With this number of the Journal we present to our readers the portrait of Dr. D. E. Salmon, the chief of the United States Bureau of Animal Industry, who has been connected with the official veterinary work of this country since Congress first instituted investigations of contagious animal diseases as provided by the appropriation of 1878.

Dr. Salmon was born in Morris county, New Jersey, July 23, 1850. He entered Cornell University at its opening in 1868, being a member of its first freshman class. Here he became acquainted with Prof. James Law, who had just come to America to fill the chair of Veterinary Science at this new institution. At that time there were few who realized the importance of veterinary instruction, or who could see any reason for including such studies among the branches taught by our higher institutions of learning.

It was the aim of Ezra Cornell, however, "to found an institution where any person could find instruction in any study," and with an admirable appreciation of the value of knowledge in regard to the diseases of animals he determined that veterinary medicine and surgery should be well represented in the faculty of the University which was to bear his name.

Very early in his course at Cornell the subject of our sketch became impressed with the importance of the position and work which were soon to be assumed in this country by educated veterinarians. Even then the flocks and herds of the land had multiplied until their numbers were enormous; and many of our States were without a single veterinarian competent to diagnose the most important animal plagues, much less to give intelligent advice for their control. Massachusetts had succeeded only two years before in ridding that State, after a six year's struggle, of an outbreak of pleuro-pneumonia, which might have been stamped out in as many months. The investigation just made by Prof. Gamgee, under the direction of the United States Department of Agriculture, had shown the existence of contagious pleuro-pneumonia from Long Island, N.Y., on the north, to the District of Columbia on the south. The most extensive outbreak of Texas fever which this country has ever known, and which resulted from the wide distribution of Southern cattle over the Northern States in 1868, was then in progress, and was attracting attention everywhere. Hog cholera, although considerably disseminated before the war, was lost sight of for a few years on account of the stirring events of the times, but in 1868 it was again attracting the attention of writers on agricultural and sanitary subjects. These diseases, widely distributed in our own land, and threatening our food supply; with foot and mouth disease and rinderpest ravaging the herds of Europe and liable to be imported at any time, gave unmistakable evidence that our National and State governments would soon appreciate the value of veterinary science.

Dr. Salmon graduated at Cornell in 1875 with the degree of Bachelor of Veterinary Science. The same year he visited Europe, and after remaining two terms at the French veterinary school at Alfort returned to America and began the practice of his profession in Newark, N. J.

In 1875, on account of impaired health, he went to Asheville, N. C., for the benefit of the southern mountain climate. In 1876 Cornell gave him the advanced degree of Doctor of Veterinary Medicine. In 1877 we find him non-resident lecturer on Veterinary Science in the University of Georgia.

The appropriation by Congress in 1878, of $10,000 for the investigation of animal diseases, led to the appointment of a number of veterinarians, including Dr. Salmon, who were directed to devote two months to the study of swine diseases. The reports of these investigations were published by the Department of Agriculture in a special volume in 1879. It was not expected that this brief investigation would solve all the problems connected with hog cholera, but it was hoped that certain leading questions, such as its nature, cause and identity in different parts of the country would be settled, and that some practical measures of prevention would be suggested. To this extent the inquiry was successful, and it was demonstrated that there was a specific, contagious disease of swine widely disseminated over the country, and that prevention could only be accomplished by controlling the spread of the contagion and destroying it wherever found.

The State of New York attempted to stamp out contagious pleuro-pneumonia in 1879, and Dr. Salmon was appointed on the veterinary staff as an assistant of Prof. Law. The spring and summer were devoted to this work, but with the exhaustion of the appropriation in the autumn, he accepted a commission from the United States Department of Agriculture to investigate animal diseases in the Southern States, and was instructed to make a specialty of Texas or Spanish fever. He then began that extensive series of studies of cattle diseases in the South which demonstrated the identity of the "distemper" of Virginia and North Carolina with the "murrain" of Georgia, Alabama, Mississippi and Tennessee, and with the Texas fever of the Northern and Western States. During the investigation he directed a careful survey of the whole country, from the Atlantic seaboard in Virginia to the Rio Grande river, accurately outlining the district permanently infected with this contagion, and showing that northern cattle taken into that district contracted the same disease that was disseminated by Texas and other Southern cattle when taken North.

In 1880 he began the investigation of fowl cholera, demonstrating that the germ found in that disease was its essential cause, that it could be made to multiply locally and act as a vaccine by simply diluting the virus to a sufficient degree. He also at this time formulated a theory of immunity from contagious diseases, based upon numerous experiments, in which were foreshadowed a new method of prevention through the use of chemical substances formed during the growth of the specific germs. This method has since been satisfactorily demonstrated by experiments made under his direction, and he is still at work upon the details which must be learned before it is put into practical operation.

Early in 1883 he was called to Washington by Commissioner Loring to establish a Veterinary Division in the Department of Agriculture. In doing this a plan was adopted for an extensive investigation of animal diseases, a laboratory for histological and bacteriological research was fitted up, and seven acres of land near the city were rented for an experimental station. Within a year Congress passed an act establishing the Bureau of Animal Industry, and Dr. Salmon was at once appointed Chief of this Bureau, a position which he still holds.

Since he has been in the Department of Agriculture a large number of investigations have been made under his direction. In 1883 he was appointed by President Arthur a member of a commission to investigate the quality of our pork products and the prevalence of trichiniasis, and his report on this subject has been received without question. In 1884 he investigated the supposed outbreak of foot-and-mouth disease in Kansas, Missouri and Illinois, and allayed apprehensions by deciding it to be ergotism. In the same year he discovered and traced out an outbreak of pleuro-pneumonia which extended to Ohio, Illinois, Kentucky and

Missouri, and succeeded by co-operating with State authorities in securing its extermination wherever found. It has since been discovered, however, that it had crept into Chicago unobserved, where it smoldered for two years and was brought to light by the State veterinarian in September, 1886. The Bureau of Animal Industry now has a force of Inspectors engaged there for its suppression.

Since its organization the Bureau of Animal Industry has made an inspection of the district from Long Island, New York to Virginia to determine the extent and prevalence of pleuro-pneumonia. In August, 1886, an agreement of co-operation for stamping out this disease was made with the State authorities of Maryland, and the slaughter of diseased animals has been going steadily on. In March of this year, an appropriation of five hundred thousand dollars was made to the Bureau, and the work extended to the slaughter of exposed as well as of diseased animals.

New and efficient rules and regulations for co-operation with State authorities were at once prepared and sent to the Governors of the various States for their acceptance; and in States where no laws existed authorizing co-operation a suitable bill was drawn and presented to the legislature for its consideration. As a result of such energetic work more than half of the States have already given the Bureau full authority to stamp out the lung plague should it be found within any of their borders, and hardly sixty days have passed since the effort was begun. This work is now in active progress in the States of New York, New Jersey, Maryland, Virginia and Illinois.

While this executive work has been pushed so successfully, the scientific work of the Bureau has not been neglected. In the report of 1885, the first accurate description was given of the germ of hog cholera and of the lesions which it produces in swine and other animals. Since then another swine disease resembling or identical with the *schweineseuche* of Schutz has been studied and will be fully described in the forthcoming report for 1886. A thorough study of the entozoa affecting the domesticated animals of the United States is also in progress, and is yielding very important results.

In addition to this work, which comes strictly within the province of the Bureau of Animal Industry as defined in the act of Congress establishing it, the quarantine of imported animals, formerly in charge of the Treasury Department, is now under the direction of Dr. Salmon.

As a recognition of Dr. Salmon's labors in behalf of Veterinary Science, the Royal College of Veterinary Surgeons of Great Britain has recently conferred upon him the degree of Honorary Associate—the highest professional honor which that body can bestow. He is also a Fellow of the American Association for the Advancement of Science, and Chairman of the Committee on Animal Diseases and Animal Foods of the American Public Health Association.

## Figures and Tables

**Figure f1:**